# IDENTIFYING THE FRAILTY COMPONENTS THAT ARE MOST IMPORTANT IN PREDICTING POSTSURGICAL OUTCOMES

**DOI:** 10.1093/geroni/igac059.1569

**Published:** 2022-12-20

**Authors:** Mamoun Mardini, Emily Smail, Patrick Tighe, Catherine Price, Christopher Kaufmann, Todd Manini

**Affiliations:** University of Florida, Gainesville, Florida, United States; University of Florida, Gainesville, Florida, United States; University of Florida, Gainesville, Florida, United States; University of Florida, Gainesville, Florida, United States; University of Florida, Gainesville, Florida, United States; University of Florida, Gainesville, Florida, United States

## Abstract

Presurgical frailty among patients is strongly associated with poor postsurgical outcomes. In the prior presentations of this symposium, we assessed the associations between each individual frailty syndromic component and three postsurgical outcomes. However, it remains unclear whether individual relationships remain after accounting for presence of the other components. In regression models that included all frailty components, demographic information, and medical history, results showed that weight loss and slow walking speed were significantly associated with length of stay and 30-day mortality, respectively. Of note, the odds of mortality in patients with slow walking speed was over three times higher than patients with normal walking speed. Lastly, weaker grip strength was strongly associated with discharge to more intensive care (vs. to home). These findings suggest that frailty screening should be universally applied before surgical procedures for better risk stratification.

